# Design a novel integrated screw for minimally invasive atlantoaxial anterior transarticular screw fixation: a finite element analysis

**DOI:** 10.1186/s13018-020-01764-w

**Published:** 2020-07-06

**Authors:** Yingkai Zhang, Cheng Li, Lei Li, Yanyan Sun, Zeqing Li, Yunli Mei, Xinyuan Feng

**Affiliations:** 1 Department of Orthopaedic Surgery, Shengjing Hospital of China Medical University, Sanhao Road 36, Shenyang City, 110001 Liaoning Province People’s Republic of China; 2 Shandong Weigao Orthopaedic Device co., Ltd., Weihai, 264300 People’s Republic of China

**Keywords:** Atlantoaxial instability, Atlantoaxial screw fixation, Atlas, Axis, Transarticular screw fixation, Finite element analysis, Biomechanics

## Abstract

**Purpose:**

To design a new type of screw for minimally invasive atlantoaxial anterior transarticular screw (AATS) fixation with a diameter that is significantly thicker than that of traditional screws, threaded structures at both ends, and a porous metal structure in the middle. The use of a porous metal structure can effectively promote bone fusion and compensate for the disadvantages of traditional AATSs in terms of insufficient fixation strength and difficulty of bone fusion. The biomechanical stability of this screw was verified through finite element analysis. This instrument may provide a new surgical option for the treatment of atlantoaxial disorders.

**Methods:**

According to the surgical procedure, the new type of AATS was placed in a three-dimensional atlantoaxial model to determine the setting of relevant parameters such as the diameter, length, and thread to porous metal ratio of the structure. According to the results of measurement, the feasibility and safety of the new AATS were verified, and a representative finite element model of the upper cervical vertebrae was chosen to establish, and the validity of the model was verified. Then, finite element-based biomechanical analysis was performed using three models, i.e., atlantoaxial posterior pedicle screw fixation, traditional atlantoaxial AATS fixation, and atlantoaxial AATS fixation with the new type of screw, and the biomechanical effectiveness of the novel AATS was verified.

**Results:**

By measuring the atlantoaxial parameters, the atlantoaxial CT data of the representative 30-year-old normal adult male were selected to create a personalized 3D printing AATS screw. In this case, the design parameters of the new screw were determined as follows: diameter, 6 mm; length of the head thread structure, 10 mm; length of the middle porous metal structure, 8 mm (a middle porous structure containing an annular cylinder ); length of the tail thread structure, 8 mm; and total length, 26 mm. Applying the same load conditions to the atlantoaxial complex along different directions in the established finite element models of the three types of atlantoaxial fusion modes, the immediate stability of the new AATS is similar with Atlantoaxial posterior pedicle screw fixation.They are both superior to traditional atlantoaxial anterior screw fixation.The maximum local stress on the screw head in the atlantoaxial anterior surgery was less than those of traditional atlantoaxial anterior surgery.

**Conclusions:**

By measuring relevant atlantoaxial data, we found that screws with a larger diameter can be used in AATS surgery, and the new AATS can make full use of the atlantoaxial lateral mass space and increase the stability of fixation. The finite element analysis and verification revealed that the biomechanical stability of the new AATS was superior to the AATS used in traditional atlantoaxial AATS fixation. The porous metal structure of the new AATS may promote fusion between atlantoaxial joints and allow more effective bone fusion in the minimally invasive anterior approach surgery.

## Introduction

Atlantoaxial instability and atlantoaxial dislocation often lead to spinal cord compression, spinal cord injury, increased rates of paraplegia, and effects on the respiratory centre, and in severe cases, it can endanger the lives of patients [1, 2]. The key to solving this problem is to fuse the atlantoaxial complex by surgical procedures so that the atlantoaxial joint can acquire a stable structure, and the nerve compression can be relieved. At present, the methods for atlantoaxial fusion can be classified as posterior atlantoaxial fusion and anterior atlantoaxial fusion. Posterior atlantoaxial fusion can achieve good biological stability and a very high fusion rate [3]; however, improper screw implantation may cause complications such as vertebral artery injury and spinal cord injury [4], and in posterior atlantoaxial fusion, the injury to the neck muscles and posterior ligament complex of the patient are considerable and may cause extensive soft tissue injury, especially in the elderly [5]. Percutaneous minimally invasive anterior transarticular screw (AATS) fixation is an effective surgical approach to address atlantoaxial instability [6, 7], and this approach results in smaller surgical wounds, less damage to muscle ligaments, and less risk than posterior atlantoaxial surgery. However, the immediate stability of anterior minimally invasive surgery is not clear, and the amount of bone graft and the position of bone grafting are not easy to control. When the amount of bone graft is too large, patients are prone to dysphagia [8], and when the amount of bone graft is too small, long-term atlantoaxial complex fusion will be affected [6]. Many studies have shown that a porous metal structure can effectively promote the bone fusion rate, which has been verified in a variety of models [9–11]. By taking advantage of this characteristic of the porous metal structure, a new type of AATS was designed, threaded structures at both ends, and a porous metal structure in the middle. The diameter of the improved AATS is significantly larger than that of the traditional AATS; thus, it can provide better immediate stability. The bone fusion-promoting characteristic of the porous metal structure can compensate for the shortcoming of the difficulty of bone fusion of traditional AATS fixation. In addition, finite element analysis was used to verify the biomechanical stability of the new AATS.

## Materials and methods

Screening criteria: (1) Patients with cervical degenerative disease and severe ligament ossification were excluded. (2) Patients with a history of trauma were excluded, and only patients with no imaging findings such as upper cervical spine fracture were included. (3) Patients with developmental deformity of the upper cervical spine were excluded, and only patients with normal physiological structure of upper cervical spine were included. (4) Patients with vertebral artery dysplasia, a high-riding vertebral artery, and other developmental abnormalities were excluded.

The original computed tomography (CT) data (DICOM format) of 113 cases of normal atlantoaxial joints meeting the study screening criteria from June 2018 to December 2018 were selected from the image database of Shengjing Hospital of China Medical University to complete and verify the rationality and safety of the device design work. Then, the atlantoaxial CT data of one typical case will be selected for the establishment of a finite element model. The review and use of the CT scanning data were approved by the institutional ethics committee.

### Measurement of the AATS path and Verify feasibility of implanting the new AATS

**Fig. 1:**
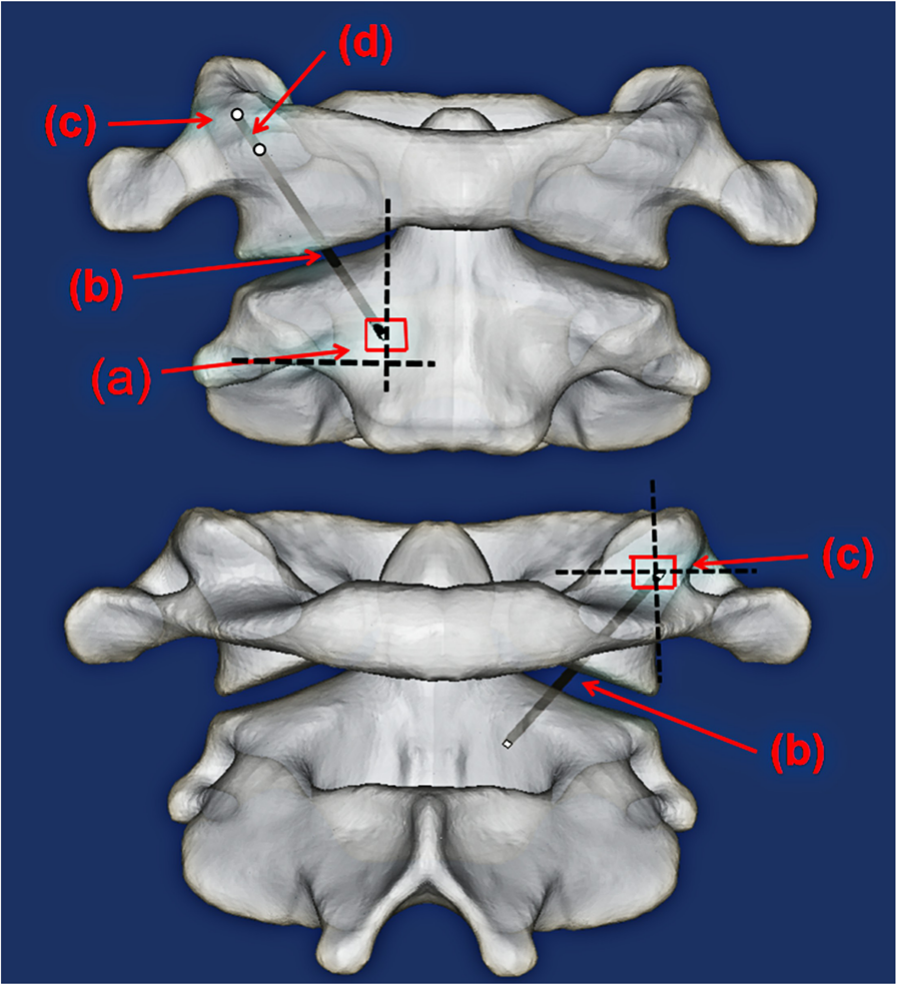
Point (**a**) in the figure shows the screw entry region, which is 4 mm above the junction between the inferior edge of the lateral arch of the axis and the lateral edge of the vertebral body of the axis. Point (**b**) shows the implantation direction of the device and (**b**) is the midpoint of the posterior edge of the superior articular process of the atlas. Point (**c**) indicates the screw path, and point (**d**) is the 5 mm safety buffer zone

**Fig. 2:**
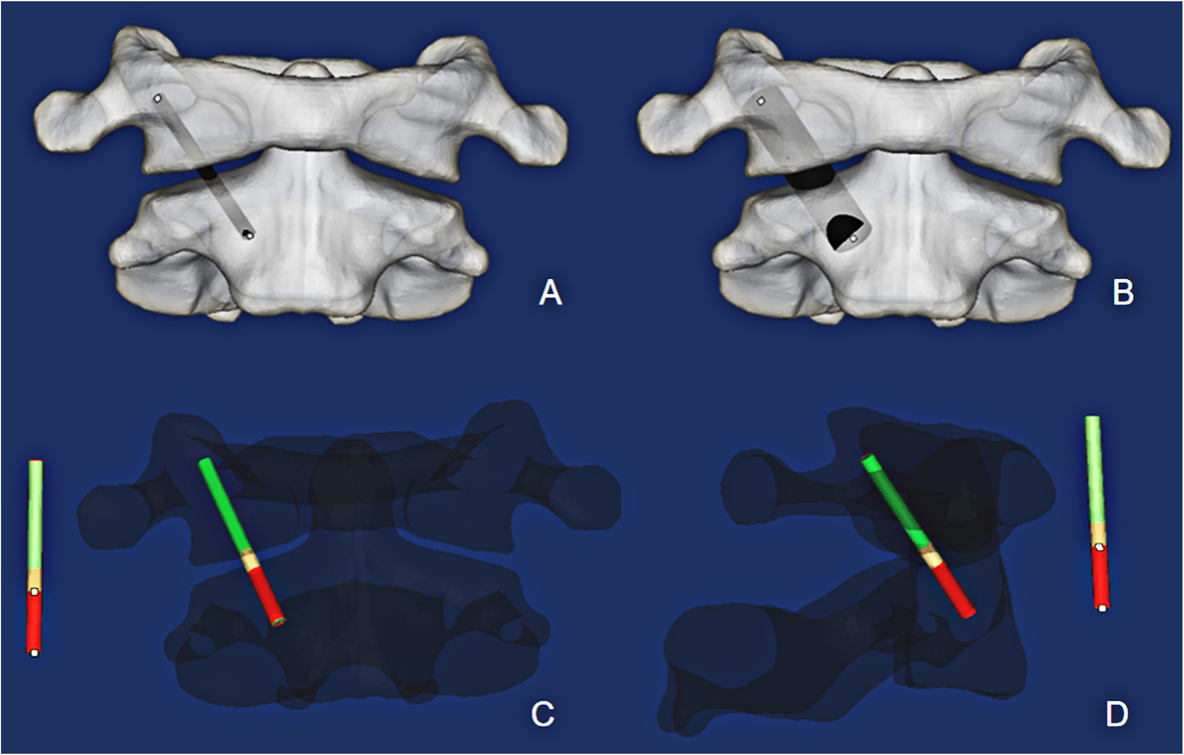
The cylinder was gradually thickened in the 3D reconstruction until it broke through the outer or inner wall of the superior articular process of the atlas (**a**, **b**), and this diameter of the cylinder was recorded. The implantation of the cylinder was simulated during the 3D reconstruction. The red portion is the length of the cylinder in the lateral mass of the axis, the yellow portion is the length of the cylinder in the atlantoaxial joint space, and the green portion is the length of the cylinder in the lateral mass of the atlas (**c**, **d**)

The new AATS is divided into three parts: the head contains a threaded structure, the middle section has a porous titanium alloy metal structure (with a solid cylinder inside to reinforce the strength of the device), and the tail has a threaded structure. Surgical operations were simulated in the Mimics 19.0(Materialize Inc., Leuven, Belgium) software to determine a proper entry point and an implantation direction of the new AATS (a cylinder was used to simulate the screw). The optimal screw diameter was designed through measurement of the distance L from the screw entry point (4 mm above the junction between the inferior edge of the lateral arch of the axis and the lateral edge of the vertebral body of the axis) to the posterior edge of the superior articular process of the atlas. Placing the screw in the above implantation direction can maximise the use of the space of the lateral mass of the atlas [12, 13] (see Fig. 1) because when the posterior edge of the superior articular process of the atlas is close, the available space is small. A length of 5 mm was reserved as a safety buffer zone, and the total length of the device was recorded as (L-5) mm. At this time, the diameter of the device was increased until it broke through the external or internal wall of the superior articular process of the atlas. The length of the screw in the lateral mass of the axis, lateral mass of the atlas, and atlantoaxial articular process space was measured (See Fig. 2).

### Validation of the finite element model of the upper cervical vertebrae

According to the result of the measurement, the atlantoaxial CT data of a 30-year-old normal adult male were finally selected for the establishment of a finite element model. The CT data of the lower part of the occipital bone, atlas, and axis of an adult male were imported into the Mimics software for three-dimensional (3D) reconstruction. After the 3D model was subjected to smoothing and mirroring treatment, the primary geometry was embedded into Geomagic Studio 12.0 (3D System Corporation, Rock Hill, South Carolina, USA) to construct a symmetrical model.Next, it was imported into the Hypermesh12.0 (Altair, Troy, MI, USA) software for relevant preprocessing, and tetrahedral meshing was performed for the lower part of the occipital bone, atlas, and axis models according to the cancellous and cortical bone of the human skeleton. The FEA model was assembled with material properties of the various constituent components as summarized in Table 1. Furthermore, major ligaments of cervical spine were also incorporated in the model with the insertions of various ligaments to vertebrae determined from anatomic text [18]. Most of the ligaments were modelled using two-node nonlinear link elements, which only permit tensile axial force transmission. Sliding contact definitions with friction were used for the facet joints as well as between the occiput and the atlas, the atlas and the dens, the dens and the transverse ligament, and the atlas and the axis, and coefficient of friction is defined as 0.1 [16].

**Table 1:** Material properties used for various components in the finite element model [14–17]

Components	Poisson’s ratio	Young’s modulus (MPa)	cross-sectional area(mm^2^)	Reference
Cortical bone	0.2	15000	–	[14]
Cancellous bone	0.2	500	–	[14]
Anterior longitudinal ligament	0.3	30	6	[15]
Posterior longitudinal ligament	0.3	20	5	[15]
Inter spinous ligament	0.3	10	5	[15]
Ligamentum flavum	0.3	10	5	[15]
Alar ligament	0.3	5	22	[15]
Transverse ligament	0.2	20	–	[15]
Nuchal ligament	0.2	20	10	[15]
Supraspinous ligament	0.3	10	10	[15]
Capsular ligament	0.3	10	46	[16]
Screw and rod	0.3	110000	–	[17]

#### Stable atlantoaxial complex

**Fig. 3:**
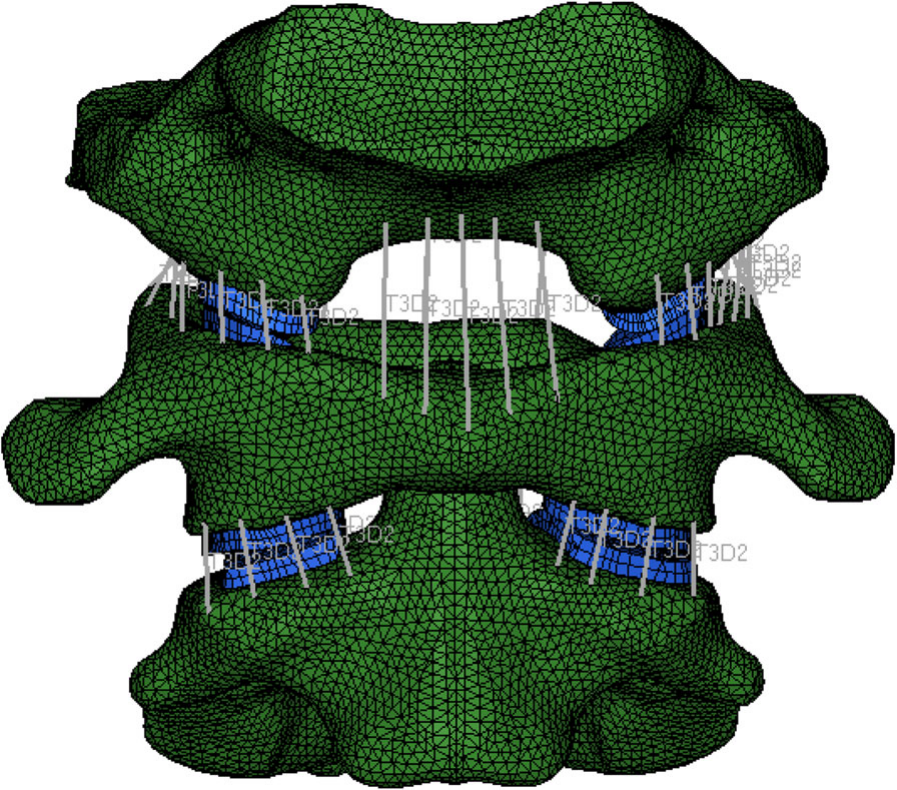
Stable atlantoaxial complex

In the occipital bone, which was composed entirely of cancellous bone, 7360 nodes and 69,726 units were established. The atlas and axis were internally composed of cancellous bone and externally covered with 1.5 mm of cortical bone, for which 15,069 nodes and 60,780 units were established for the atlas, and 17,069 nodes and 70,195 units were established for the axis (Fig. 3).

All of the abovementioned finite element models were imported into Abaqus 6.11 (Dassault Systems Corporation), and boundary conditions were created for the above models. Starting from the initial analysis step, the type of analysis step for displacement and the rotation angle was created, and the degrees of freedom in various directions for all elements at the bottom of the axis were limited. A concentrated, vertically downward force of 40 N was applied from both sides of the occipital bone to simulate the gravity-induced effects of the human head. A moment of 1.5 Nm was applied to the model occiput along each sagittal plane, coronal plane, and cross-section, and the left and right axial rotation, flexion, extension, left and right lateral bending of the skull base (C0), the first cervical vertebra (C1), and the second cervical vertebra (C2, the axis) were observed. The measured data were compared with the in vitro model of Panjabi et al. [19, 20] to verify the accuracy of the model, and the left and right axial rotation, flexion, extension, and lateral bending between C1 and C2 under normal physiological loading conditions in the three fusion modes were recorded.

### Comparison of the stability of the three atlantoaxial fusion methods

**Fig. 4:**
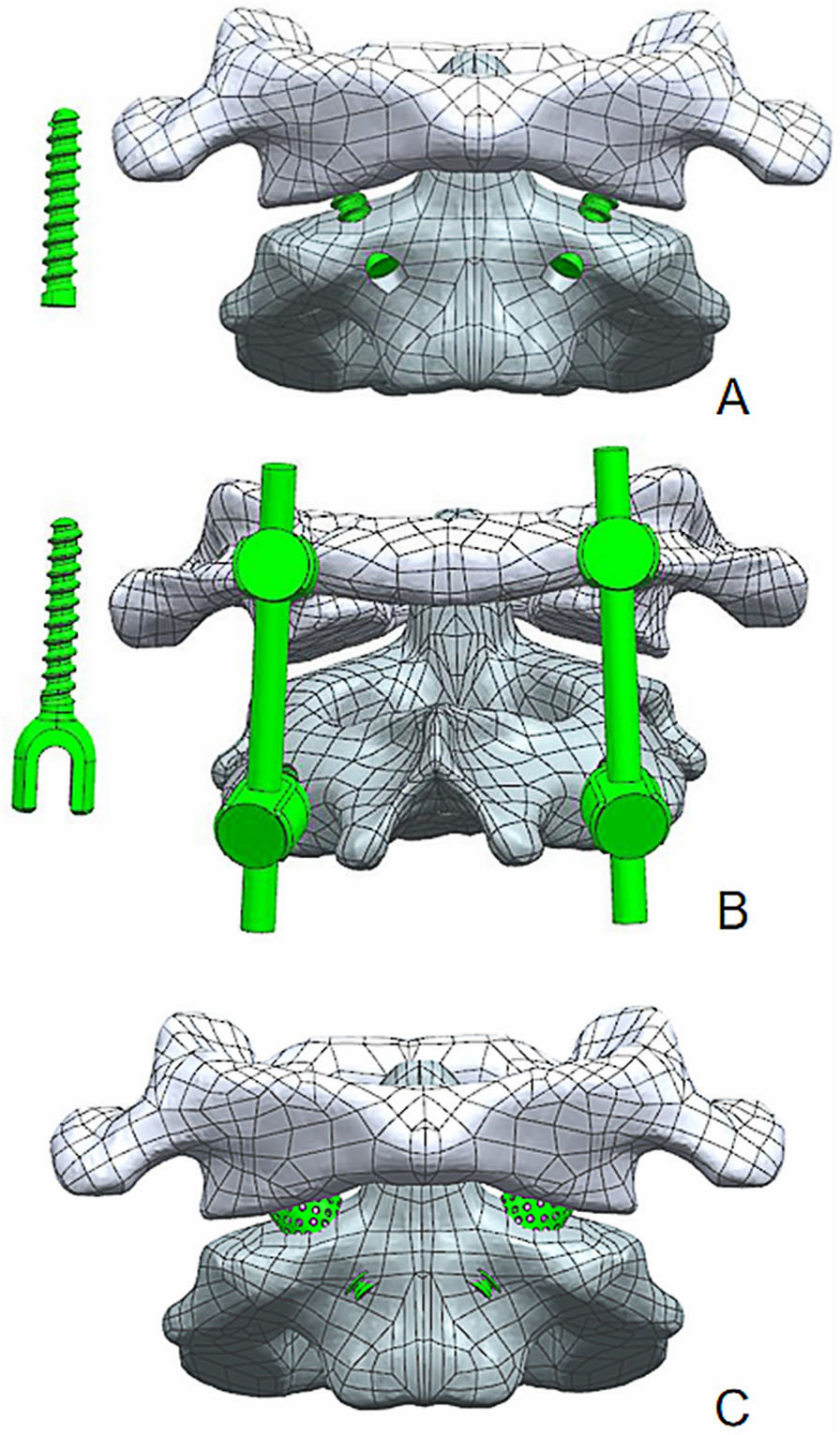
Development of an atlantoaxial fusion method model. **a** Traditional atlantoaxial anterior transarticular screw fixation. **b** Atlantoaxial posterior pedicle screw fixation. **c** New AATS

1. Traditional atlantoaxial posterior pedicle screw fixation: specification and modelling of the screws (diameter 3.5 mm). To simulate posterior atlantoaxial fusion, the atlantoaxial pedicle screw system was used to fix the unstable atlantoaxial complex through the atlantoaxial pedicle and destroy the posterior ligament structure. A total of 1938 nodes and 24,845 units were built in the assembled screw and rod system (Fig. 4).
2. Traditional atlantoaxial AATS fixation: Specification and modelling of the screws (diameter 3.5 mm). To simulate AATS fixation, an atlantoaxial anterior AATS was used to fix the unstable atlantoaxial complex via the atlantoaxial articular process and destroy the anterior joint capsule. A total of 2425 nodes and 21,475 units were established by the assembled screws (Fig. 4).
3. Fusing the atlantoaxial complex using the new AATS (new atlantoaxial AATS fixation): Specification and modelling of screws (diameter 6 mm). To simulate AATS fixation, the new AATSs were used to fuse the atlantoaxial complex and destroy the anterior joint capsule. The diameter of the new AATS was 6 mm (the middle porous structure contains a solid cylinder with an outer diameter of 3 mm and an inner diameter of 3 mm, and a cylinder which removes multiple spheres of 600 μm diameter is used to simulate a porous structure, and the porosity of cylinder is 30%), and a total of 167 nodes and 5229 units were established (Fig. 4).

To more directly compare the stability of the three atlantoaxial fusion modes, a concentrated moment of normal physiological load (1.5 Nm) was applied. The atlantoaxial left and right axial rotation was observed by applying a moment of normal physiological load (1.5 Nm) along the horizontal direction of the posterior arch of the atlas of the three models. Atlantoaxial flexion and extension were observed by applying a moment of normal physiological load (1.5 Nm) along the direction perpendicular to the posterior arch of the atlas. Atlantoaxial lateral bending was observed by applying a moment of normal physiological load (1.5 Nm) along the direction perpendicular to the articular process of the atlas, and the degree of stability of the three fusion modes was compared. The changes in the stress concentration in the screws of the new AATS fixation and traditional anterior atlantoaxial fusion were observed through finite element analysis, and the difference between the new AATS and the AATS used in the traditional anterior fusion method was evaluated.

## Results

### Measurement results of the parameters for the new AATS

**Fig. 5:**
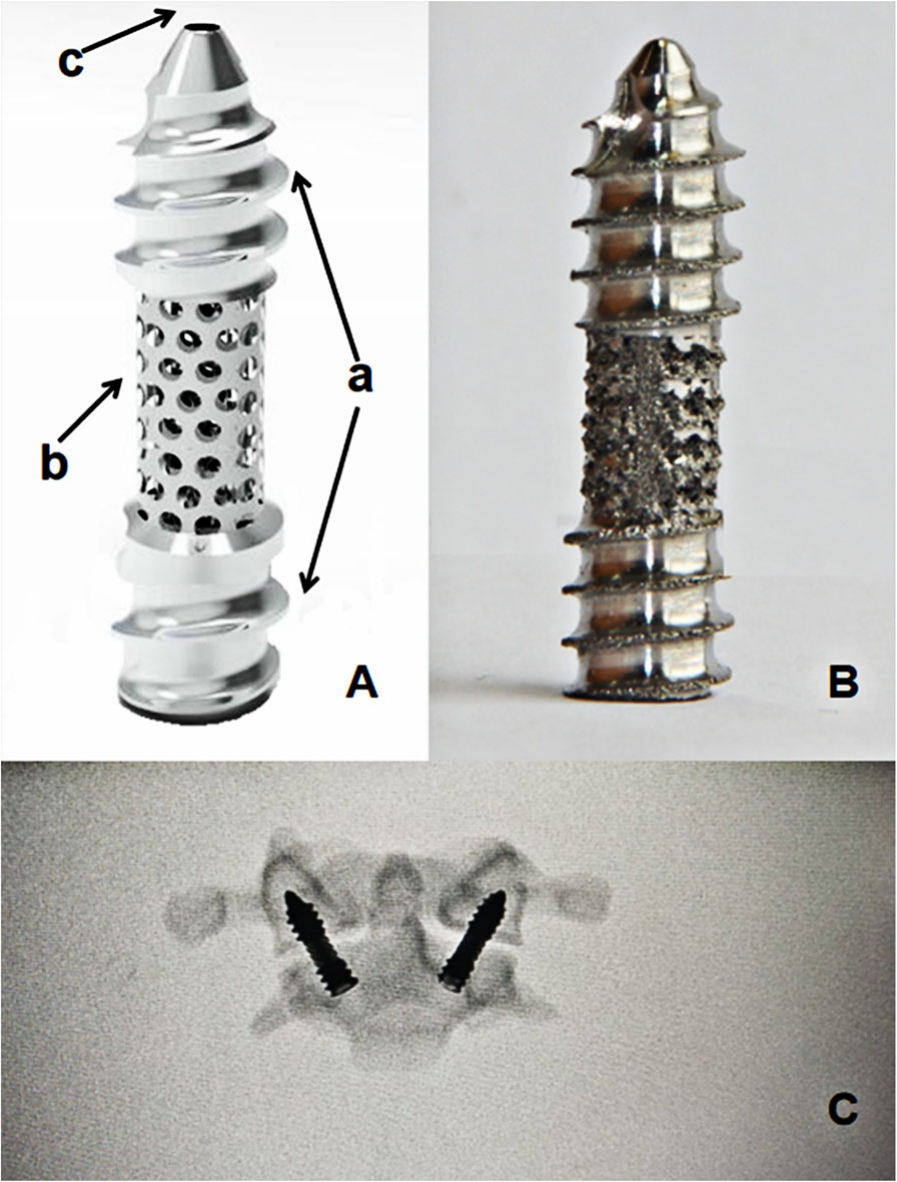
**A** New AATS Model. **a** Threaded structures at both ends. **b** Porous metal structure in the middle. **c** Internal annular solid cylinder with an outer diameter of 3 mm and an inner diameter of 1 mm (for guide wire positioning). **B** New AATS. **C** New AATS in atlantoaxial model

The axis length of new AATS (length A) was 8.5 ± 0.7 mm on the left side and 8.6 ± 0.6 mm on the right side; the length in the atlantoaxial articular process space (length B) was 6.6 ± 0.3 mm on the left side and 6.7 ± 0.4 mm on the right side; the length in the lateral mass of the atlas (length C) was 10.5 ± 0.6 mm on the left side and 10.6 ± 0.5 mm on the right side. According to the result of the measurement, the atlantoaxial CT data of a 30-year-old normal adult male were finally selected for the establishment of a finite element model (Table 2). To allow the cancellous bone to grow along the porous metal, the porous metal part needs to run through the atlantoaxial articular space and penetrates the cortical part of the inferior articular process of the atlas and the superior articular process of the axis. Therefore, when designing the screw, the lengths of the head and tail near the end of the porous structure needed to be reduced by 2 mm, which is the cortical thickness, while the length of the middle porous metal needed to be increased at both ends by 2 mm. To ensure the strength of the screw, the middle porous metal consisted of a solid cylinder structure. By measuring relevant atlantoaxial parameters and the individual differences of patients, the 3D printing technology is used to make new AATS suitable for patients. According to the design parameters, the new AATS which is suitable for this case was set as follows: diameter, 6 mm; length of the thread structure of the head, 10 mm; length of the middle porous metal, 8 mm (the middle porous structure contains a solid cylinder with an outer diameter of 3 mm and an inner diameter of 3 mm); length of the thread structure of the tail, 8 mm; and total length, 26 mm. A relevant finite element model was established for analysis (Fig. 5).

**Table 2:** Measured atlantoaxial data (unit: mm)

	Left	Right	Left(FEA data)	Right(FEA data)
Length A	8.5 ± 0.7	8.6 ± 0.6	8.6	8.6
Length B	4.1 ± 0.3	4.2 ± 0.4	4.2	4.2
Length C	12.5 ± 0.6	12.6 ± 0.5	12.6	12.6
Diameter	8.1 ± 0.4	8.2 ± 0.4	8.2	8.2

### Verification of the finite element model

The established finite element model was compared with that in the in vitro study by Panjabi et al. A moment of physiological load of 1.5 Nm was applied to the occiput in all directions, and the angles of movement in the directions of flexion, extension, lateral bending, and axial rotation were compared (see Table 3). As measured, the range of motion (ROM) of the finite element model under the six loads was similar to that of the in vitro model, and the differences in the results were acceptable considering the individual differences in the models themselves. Therefore, the established finite element model can accurately simulate the biological structure of the human atlantoaxial joint (Table 4).

**Table 3:** New ATS design data used in finite element analysis

	Value (mm)
Head	10
Middle	8
Tail	8
Diameter	6

**Table 4:** Comparison of the range of motion between the current finite element model and those of experimental studies (degree)

	Segment	Panjabi et al. [19, 20]	Model 1
Flexion	C0-C1	14.4 ± 3.2	15.21
	C1-C2	12.7 ± 3.2	13.12
Extension	C0-C1	14.4 ± 3.2	15.54
	C1-C2	10.5 ± 5.0	12.36
Lateral bending	C0-C1	5.6 ± 3.0	7.65
	C1-C2	12.6 ± 7.0	15.25
Axial rotation	C0-C1	3.3 ± 2.3	4.45
	C1-C2	37.4 ± 9.0	41.95

### Axial displacement of the model

**Fig. 6:**
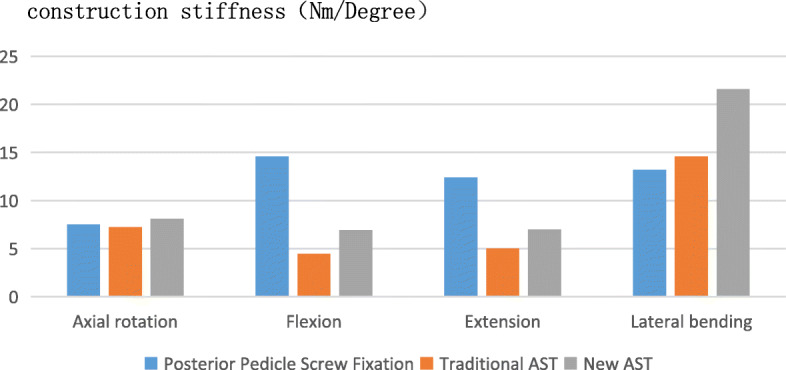
Graphical representation of the atlantoaxial fixation stiffness during flexion, extension, lateral bending, and axial rotation

The changes in the angles of axial rotation, flexion-extension, and lateral bending of the three models under the same load were compared, and all three models had a stable structure under normal physiological load. However, under the normal physiological load condition, the atlantoaxial angles changed in the directions of axial rotation, flexion-extension, and lateral bending (Table 4). The construction stiffness of the models was then calculated. The construction stiffness of the atlantoaxial posterior fusion was recorded as A, the construction stiffness of the traditional atlantoaxial anterior fusion was recorded as B, and the construction stiffness of the new AATS was recorded as C. Under the same loading conditions, comparisons of the data showed the following results: in terms of axial rotation, C > A > B; in terms of flexion, A > C > B; in terms of extension, A > C > B; and in terms of lateral bending, C > B > A (Fig. 6).

### Von Mises stress of the screw

**Fig. 7:**
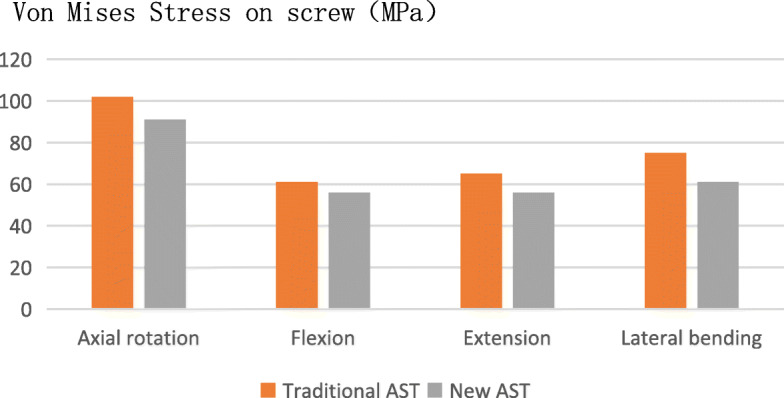
Von Mises stress on pedicle screw

When the local maximum stress on the head of the screw was compared between the traditional anterior atlantoaxial fixation and the new AATS, during axial rotation, the local maximum stress on the head of the new AATS was 56 MPa, while that on the head of the traditional anterior atlantoaxial fixation screw was 61 MPa, resulting in an 8% reduction; during extension, the local maximum stress on the head of the new AATS was 91 MPa, while that on the head of the traditional anterior atlantoaxial fixation screw was 102 MPa, resulting in a 10% reduction; during flexion, the local maximum stress on the head of the new AATS was 61 MPa, while that on the head of the traditional anterior atlantoaxial fixation screw was 75 MP, resulting in an 18% reduction; during lateral bending, the local maximum stress on the head of the new AATS was 56 MPa, while that on the head of the traditional anterior atlantoaxial fixation screw was 65 MPa, resulting in a 14% reduction (Fig. 7).

## Discussion

The surgical treatment of atlantoaxial disorders is a high-risk area in spinal surgery [21]. Atlantoaxial instability and atlantoaxial dislocation often lead to severe spinal cord injury, and in severe cases, they can endanger the lives of patients. The key to solving this problem is to fuse the atlantoaxial complex by surgery [22] so that the atlantoaxial structure can be stabilised and nerve compression can be relieved.

At present, the atlantoaxial fusion methods widely used in and outside China are classified as atlantoaxial posterior surgery and atlantoaxial anterior surgery. Atlantoaxial posterior surgery was first proposed by Gallie et al. [23] in 1939, and it has become the current method of atlantoaxial posterior pedicle screw fixation due to its continuous development and innovation [24]. This surgical procedure is a commonly used method for atlantoaxial fusion with a relatively mature technique and reliable stability. However, this surgical procedure is difficult and involves high risks, which are unavoidable for many spinal surgeons. Posterior surgery is prone to damage of the vertebral artery and nerves [25], resulting in severe consequences such as massive haemorrhage of the vertebral artery, cerebral infarction and hemiplegia caused by vertebral artery embolism, and dyspnoea resulting from central nervous system damage; in severe cases, the life of the patient may be in danger [26, 27]. Some cases involve difficulties and risks when using the posterior approach, such as the abnormal development and pathway of the vertebral artery [28–30] and congenital or iatrogenic absence of the bony structure of the atlantoaxial vertebra pedicle [31, 32], and the atlantoaxial complex can be more safely fused through the anterior approach.

The atlantoaxial anterior approach is divided into atlantoaxial transoral fusion and fixation [33, 34] and atlantoaxial transarticular fusion [13]. Atlantoaxial anterior transoral fusion and internal fixation can better expose the atlantoaxial joint and provide a better surgical field, but its disadvantages are also obvious, including the inability to perform normal orotracheal intubation and the high susceptibility to postoperative infection [35, 36]. In severe cases, a patient’s life could be in danger; therefore, the surgeon should use atlantoaxial anterior transoral fusion and internal fixation with caution. Traditional atlantoaxial anterior transarticular fusion was first proposed by Lesion et al. [37] in 1987; subsequently, some scholars continuously developed and perfected open AATS fixation [38], and all achieved good therapeutic results. Fong et al. [39] reported an anatomical study of minimally invasive surgery of the upper cervical vertebrae and demonstrated the feasibility of anterior minimally invasive surgery for upper cervical vertebrae. Wang et al. completed seven cases of minimally invasive AATS fixation [6], and except for two patients with transient dysphagia due to difficulty in controlling the amount and position of the bone graft, the remaining patients achieved good therapeutic results, confirming the feasibility of the minimally invasive AATS surgical approach. Minimally invasive AATS fixation does not damage any of the important muscles and ligaments of the cervical vertebrae, causes less trauma to the patient, and results in less intraoperative bleeding than posterior screw and rod fusion and fixation, and it preserves the cervical vertebral posterior ligamentous complex and greatly increases the stability of the cervical vertebrae. The volume of the atlantoaxial lateral mass is large, and it is not easy for the AATS to penetrate this mass, thus injury to the vertebral artery and central nervous system can occur [40, 41]. Its disadvantage is that the immediate stability during atlantoaxial fusion by anterior minimally invasive surgery is still unclear. Moreover, it is not easy to control the amount and position of the bone graft. If the bone graft amount is too large, it tends to wear the oesophagus and causes dysphagia and other discomfort; if the amount is too small, unstable fusion or even fusion failure tends to occur [6, 8]. In some cases, the efficacy of primary implantation and fusion is not certain, and a secondary surgery is required for treatment [42] and not only increases the risk of the surgery but also increases the cost of patient treatment. A study by Kim et al. [43] showed that the biomechanical stability of traditional AATS fixation was poor during flexion and extension. We performed relevant measurements of the screw path of AATS fixation, and the results showed that the AATS path can safely accommodate screws with a diameter of 6–7 mm, and that increasing the diameter of screws can improve the stability of fixation. The measurement results demonstrate that when a point at 4 mm above the junction between the inferior edge of the lateral arch of the axis and the lateral edge of the vertebral body of the axis is used as the entry point and the posterior edge of the superior articular process of the atlas is used as the implantation direction, the space of the lateral mass of the atlas can be used most effectively [12, 13]. This technique can be safely used in anterior minimally invasive surgery for most normal atlantoaxial structures. By increasing the diameter of the screw, we increased the ability of the screw to immediately stabilise the atlantoaxial complex, while the porous metal facilitated bone fusion, thus long-term atlantoaxial bony fusion could be achieved.

In recent years, many studies have shown that a porous metal structure can effectively facilitate the occurrence of bone fusion, and in spinal surgery, porous metal has been shown to fuse the vertebral body to achieve good stability and thus achieve the purpose of the surgery [9, 44–46]. Based on the above information, we designed a new AATS. Both ends of the screw contain a threaded structure, and the middle part contains a porous metal structure. The design of the porous metal structure can increase cell adhesion and promote cell differentiation, and the porous metal structure can also facilitate bone ingrowth so that bone fusion can be completed without the implantation of autologous or allogeneic bone, and related complications in bone harvesting area can be reduced [11, 47, 48]. The pore size of the porous metal is 150 μm. Some scholars have shown [49] that such a porous metal structure can not only achieve the best strength but can also fully facilitate bone ingrowth. After bone growth, a firm and stable structure is formed between the bones; this formation is similar to a “reinforced concrete” structure and can significantly increase the stabilisation effect [50–52]. Following continuous research by scholars, the processing technology of porous metals has been improved, and the osteoconductive of porous metals has been enhanced [10, 53], enabling the integration of anterior atlantoaxial transarticular internal fixation and fusion.

To increase the mechanical strength of the screws, the outer structure of the middle part of the new AATS consists of porous titanium, and the inner part is a solid cylinder with a diameter of 3 mm. We performed a biomechanical analysis by establishing a finite element model of the upper cervical vertebrae and preliminarily compared the effects of the three internal fixation methods on the stability of the atlantoaxial model and the stress distribution of the screw. The establishment of the finite element model of the upper cervical vertebrae was proposed by Puttlitz et al. [54], and Brolin et al. [16] and Zhang et al. [55] improved the model. The current model included bones and ligaments, but muscles are not included in the current model. Although muscles are assumed to be capable of stabilizing the spinal column in vivo, they are seldom tested in vitro. When their study was compared with the in vitro model of the upper cervical vertebrae developed by Panjabi et al. [19, 20], the rotation angles of flexion, extension, lateral bending, and axial rotation were the same, and the stress on the vertebral body was the same when a physiological load of 1.5 Nm was applied, validating the effectiveness of the finite element model of the upper cervical vertebrae. Therefore, it can be used clinically to replace the in vitro experiment. Several scholars have completed biomechanical analysis of cervical spine by using the above model and obtained reliable results [17, 56, 57]. Within the limits of the model, the results of finite element-based mechanical analysis showed that the immediate stability of the new AATS after implantation was superior to that of the traditional atlantoaxial posterior screw. In terms of atlantoaxial rotation and l, it can be seen that immediate fixation stability plays an important role in long-term bony fusion lateral bending, and the stability of the new AATS was superior to that of the atlantoaxial posterior screw; whereas in terms of flexion and extension, the stability of the atlantoaxial posterior screw was superior to the new AATS. This result is also the same as Kim et al. [43]. A review shows that the most commonly encountered perioperative complications were related to mechanical failure with rates as high as 7% during occipitocervical fusion and 6.7% during atlantoaxial fusion [58]. In addition, instability of implantation may cause failure of atlantoaxial fusion [59], and some scholars believe that the biomechanical stability of anterior atlantoaxial fixation is insufficient. In order to achieve long-term bony fusion, they performed one-stage anterior release and then posterior instrumentation and fusion [60, 61]. It can be seen that immediate fixation stability plays an important role in long-term bony fusion, and the more stable internal fixation, the better the outcome [62]. When compared with the stability of traditional atlantoaxial anterior screw fixation, the stiffness of the new AATS fixation was superior in terms of flexion, extension, lateral flexion, and rotation. Therefore, it improves the immediate stability of atlantoaxial and provides a better condition for the ultimate fusion of atlantoaxial and porous metals**.**Within the limits of the model, the results show that the increased diameter can not only increase the immediate stability, reduce the local stress of the screw, and reduce the possibility of screw withdrawal and deformation, but it can also increase the area of bone ingrowth into the porous metal, which is expected to achieve better fusion effects.

There are still some limitations of this study. In this study, only the computer software and atlantoaxial model were used to simulate the implantation of the new ATS in the selected cases in order to prove its feasibility and safety. We need to implant the new AATS into the cadaver model to further verify its safety. The finite element analysis only simulated the stress and displacement of the atlantoaxial model after force was applied, and the results still need to be verified by animal models. In the subsequent experiments, the device will be implanted into animal models to verify its feasibility in fusing the atlantoaxial complex.

## Conclusion

The new AATS can make full use of the atlantoaxial lateral mass space and increase the stability of fixation. Finite element analysis confirmed that the biomechanical stability of the new screw was superior to traditional atlantoaxial anterior transarticular internal fixation; the porous metal structure of the new AATS could effectively facilitate bone graft fusion between atlantoaxial joints, making the bone fusion effect of minimally invasive anterior surgery more likely.

## Data Availability

Yes

## Abbreviation

AATS: Atlantoaxial anterior transarticular screw
